# Indeterminate Lymph Nodes Assessment in Oral Squamous Cell Carcinoma Using CT, MRI, and PET-CT: A Retrospective Study

**DOI:** 10.3390/jcm15041610

**Published:** 2026-02-19

**Authors:** Jihye Ryu, Su-Yi Sim, Jae-Yeol Lee

**Affiliations:** 1 Department of Oral and Maxillofacial Surgery, Pusan National University Dental Hospital, Yangsan 50612, Republic of Korea; ryujh@umich.edu (J.R.); la_vender@pusan.ac.kr (S.-Y.S.); 2 Department of Oral and Maxillofacial Surgery, Dental and Life Science Institute & Dental Research Institute, School of Dentistry, Pusan National University, Yangsan 43241, Republic of Korea

**Keywords:** diagnostic imaging, lymph nodes, carcinoma, squamous cell of the oral cavity

## Abstract

**Objective:** This study aimed to evaluate and compare the diagnostic performance of computed tomography (CT), magnetic resonance imaging (MRI), and positron emission tomography-computed tomography (PET-CT) in detecting metastatic cervical lymph nodes in patients with oral squamous cell carcinoma (OSCC), with a particular focus on radiologically indeterminate lymph nodes. **Materials and Methods:** A retrospective analysis was conducted on OSCC patients who underwent CT, MRI, and PET-CT imaging prior to surgery, followed by histopathologic confirmation. Lymph nodes were categorized as metastatic, indeterminate, or benign based on imaging criteria specific to each modality. Diagnostic accuracy, sensitivity, and specificity were calculated using histopathology as the reference standard. **Results:** After excluding lymph nodes classified as indeterminate on preoperative imaging, CT demonstrated an accuracy of 83.6%, sensitivity of 51.9%, and specificity of 92.4% for metastatic lymph node detection. MRI and PET-CT showed comparable accuracies (81.6% and 80.8%, respectively) and identical sensitivities (53.9%). In contrast, among radiologically indeterminate lymph nodes, most lesions identified on CT and MRI were histopathologically benign, whereas PET-CT yielded very few indeterminate nodes, all of which were benign. For benign node identification, PET-CT exhibited the highest sensitivity (86.8%) but lower specificity (55.8%), while CT and MRI demonstrated more balanced specificity (73.1%) with lower sensitivity. Logistic regression confirmed SUVmax as a significant predictor of malignancy (*p* < 0.001; odds ratio 1.71, 95% CI: 1.48–2.35), and ROC analysis demonstrated strong discriminative performance (AUC = 0.88), with an optimal SUVmax cutoff of 3.6. **Conclusion:** While CT remains highly specific, PET-CT offers greater sensitivity in detecting benign and indeterminate lymph nodes, making it a valuable adjunct in preoperative assessment. SUVmax serves as a strong quantitative indicator for metastatic involvement. A multimodal imaging approach may enhance diagnostic accuracy, particularly in cases where lymph nodes lack definitive radiologic features.

## 1. Introduction

Upon diagnosis of oral squamous cell carcinoma (OSCC), a series of diagnostic evaluations is further proceeded to determine the extent and staging of the lesion, assess operability, and guide precise treatment planning. OSCC frequently involves regional lymphatic spread, which significantly influences staging and prognosis. In head and neck malignancies such as OSCC, imaging modalities play a crucial role in evaluating regional nodal metastasis. Even in early-stage OSCC, cervical lymph node metastases are present in approximately 23% of cases, and up to 40% of the body’s lymph nodes reside in the cervical region [1,2]. The identification of nodal metastases prior to surgical intervention is essential for accurate staging and optimizing surgical management. Therefore, the preoperative detection and differentiation of indeterminate lymph nodes—distinguishing benign from metastatic—is of significant clinical importance. A variety of imaging modalities—including computed tomography (CT), magnetic resonance imaging (MRI), and 18F-fluorodeoxyglucose positron emission tomography/computed tomography (PET-CT)—are routinely used to evaluate nodal status in head and neck cancers [1,3]. Meta-analyses have shown that PET-CT typically exhibits the highest sensitivity but lower specificity, while CT and MRI tend to be more specific but less sensitive [1,4]. For instance, Alsibani et al. reported that PET-CT demonstrates a sensitivity of approximately 74.5% and specificity of 83.6% in clinically N0 necks [5] whereas combined CT and MRI offer high specificity but may miss micro-metastases [4,5,6]. Indeterminate lymph nodes which lack definitive radiologic features of metastasis, present a diagnostic challenge due to their ambiguous characteristics. Even with the use of multiple imaging modalities—such as CT, MRI, and PET-CT—their relative diagnostic accuracy in differentiating lymph node pathology has yet to be conclusively established [4,7,8]. The purpose of this study is to evaluate the accuracy, specificity, and sensitivity of CT, MRI, and PET-CT, and to compare the diagnostic performance of each modality in differentiating lymph node status in patients with OSCC.

## 2. Materials and Methods

### 2.1. Study Design and Subjects

**Figure 1:**
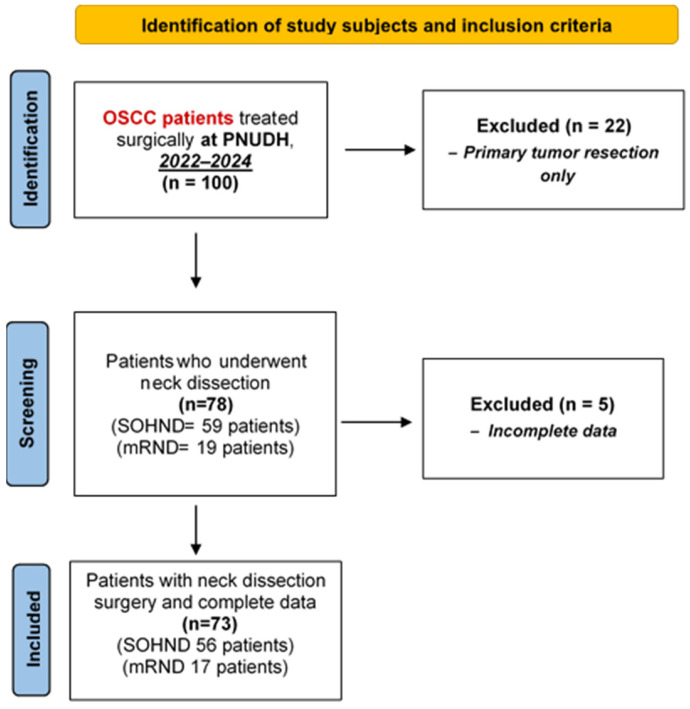
Flowchart of Patient Selection. Abbreviations: OSCC, oral squamous cell carcinoma; PNUDH, Pusan National University Dental Hospital; SOHND, supraomohyoid neck dissection; mRND, modified radical neck dissection.

This retrospective study initially identified 100 patients diagnosed with oral squamous cell carcinoma (OSCC) between 2022 and 2024 who underwent surgical treatment at Pusan National University Dental Hospital (PNUDH). Of these, 78 patients underwent neck dissection and had complete preoperative CT, MRI, and PET-CT imaging available for analysis. Inclusion criteria were: (1) histopathologically confirmed primary OSCC based on incisional biopsy, (2) availability of preoperative CT, MRI, or PET-CT imaging, and (3) histopathological confirmation following cervical lymphadenectomy. Exclusion criteria comprised 22 patients who underwent primary tumor resection without neck dissection and 5 patients with incomplete imaging records (Figure 1).

Lymph nodes (LNs) were classified as metastatic, indeterminate, or non-metastatic based on imaging interpretations provided by board-certified radiologists or nuclear medicine specialists. The diagnostic criteria varied according to the imaging modality. For CT, metastatic lymph nodes were identified based on the following features: size ≥ 15 mm in the shortest axial diameter; morphological characteristics such as necrosis, cystic change, calcification, or hyper-enhancement; irregular or ill-defined margins suggesting extracapsular spread; and asymmetric nodal distribution or the presence of three or more contiguous lymph nodes [9]. On MRI, metastatic involvement was suggested by low signal intensity on T1-weighted images and high signal intensity on T2-weighted images [9]. For PET-CT, a maximum standardized uptake value (SUVmax) of ≥2.5 was used as the threshold to suggest metastatic involvement. The classification outcomes served as the basis for further analysis of diagnostic performance and correlation with histopathological findings (Table 1). For further analysis, lymph nodes that were classified as indeterminate on preoperative CT, MRI, and PET-CT were correlated with histopathological results to evaluate the diagnostic distribution across imaging modalities.

For PET-CT, a standardized uptake value (SUVmax) threshold of ≥2.5 was used to indicate suspected metastatic involvement, as supported by prior studies [4,10]. This served as the initial classification criterion, whereas an optimal cutoff value was later derived using ROC analysis.

### 2.2. Statistical Analysis

All statistical analyses were processed using SPSS version 23.0 software (SPSS Inc, Chicago, IL, USA) Continuous variables were tested for normality using the Shapiro–Wilk test and compared using the independent *t*-test. Categorical variables were analyzed using the chi-squared test. To evaluate the independent predictive value of SUVmax, a binary logistic regression model was constructed with metastasis as the outcome variable. Receiver Operating Characteristic (ROC) analysis was conducted to assess the diagnostic performance of SUVmax and identify an optimal cutoff value using the Youden index. Diagnostic accuracy, sensitivity, and specificity of CT, MRI, and PET-CT were calculated using histopathological findings as the reference standard based on the initial radiologic classification of benign and metastatic lesions. In cases where lymph nodes were indeterminate on CT or MRI, the additional diagnostic value of PET-CT was assessed. To analyze the statistical relationship between radiologic findings and pathological outcomes, a logistic regression analysis was conducted, particularly focusing on the association between SUVmax values and histopathologic diagnosis. Group comparisons for continuous variables were performed using the independent *t*-test, while the chi-squared test was applied for categorical data to evaluate associations between imaging characteristics and nodal pathology. Statistical analyses were performed using a significance level of *p* < 0.05.

## 3. Results

Analysis of lymph node characteristics on CT images across benign (n = 164), indeterminate (n = 46), and metastatic (n = 43) groups revealed distinct distribution patterns (Table 2). Multifocality was predominantly associated with metastatic and indeterminate LNs, with multiple lesions observed in 55.8% of indeterminate and 32.6% of metastatic cases, compared to only 12.8% in benign LNs. An unclear nodal boundary was identified in only one metastatic case and was absent in all others. No microcalcifications were detected across all groups. Cystic change was observed in two metastatic LNs and none of the others. Necrotic change was notably more common in metastatic LNs (23.3%) versus benign (0.6%) and indeterminate (0%). An L/T (long-to-short axis) ratio >2 was more frequent in benign LNs (6.1%) compared to indeterminate (8.7%) and metastatic (7.0%) groups.

MRI was evaluated across benign (n = 164), indeterminate (n = 38), and metastatic (n = 43) lymph node (LN) groups (Table 3). Multifocality was observed more frequently in indeterminate LNs (55.3%) and metastatic LNs (20.9%) than in benign LNs (12.2%). An unclear nodal boundary was identified only in metastatic LNs (4.7%) and was absent in both benign and indeterminate groups. Microcalcification was not observed in any group. Cystic change was rare, detected in a single benign LN (0.6%) and absent in indeterminate and metastatic LNs. Necrotic change was exclusively present in metastatic LNs, observed in 16.3% (7/43), and was not identified in benign or indeterminate LNs. Overall, multifocality and necrotic change were more frequently associated with metastatic involvement.

Total of 244 lymph nodes were identified during specimen sectioning following neck dissection and subsequently evaluated histopathologically. Among these, 192 lymph nodes were confirmed as benign, while 52 were diagnosed as metastatic.

Regarding nodal level distribution, levels I and II were the most commonly involved sites in both groups, each accounting for 42.3% of benign and metastatic nodes. Level III involvement was notably more frequent in benign LNs (35.9%) compared to metastatic LNs (7.7%). Levels IV and V were infrequently involved in both groups(Table 4).

### 3.1. Diagnostic Performance

**Figure 2:**
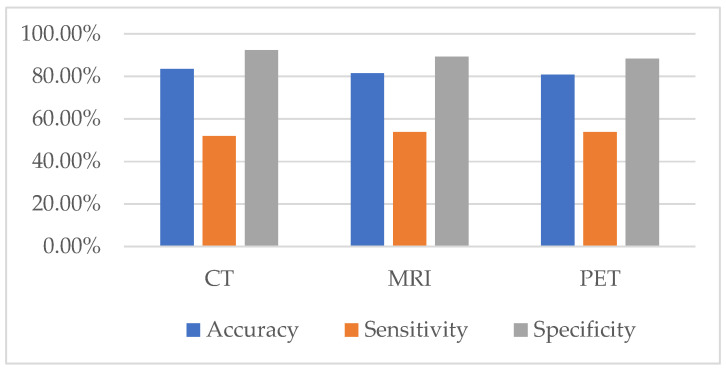
Accuracy, Sensitivity, Specificity of CT/MRI/PET-CT: the highest specificity is 92.39% while MRI showed slightly higher sensitivity (53.85%). Overall diagnostic accuracy was comparable among modalities, ranging from 80.8% to 83.6%.

Based on the initial radiologic assessment of metastatic and benign lesions, CT demonstrated the highest diagnostic accuracy (83.6%) and specificity (92.39%) for detecting metastatic disease, although its sensitivity was relatively limited (51.92%). MRI showed a comparable accuracy of 81.6%, with a slightly improved sensitivity (53.85%) and moderately high specificity (89.34%). PET exhibited similar diagnostic accuracy (80.8%) and sensitivity (53.85%) to MRI, though with slightly reduced specificity (88.32%). For benign disease prediction, PET achieved the highest accuracy (80.32%) and sensitivity (86.80%), indicating its strength in correctly identifying non-metastatic cases. However, this was accompanied by lower specificity (55.77%) compared to MRI and CT. Both CT and MRI performed similarly in predicting benign cases, with CT demonstrating an accuracy of 74.30% (sensitivity 74.62%, specificity 73.08%) and MRI showing an accuracy of 74.70% (sensitivity 75.13%, specificity 73.08%) (Table 5, Figure 2).

Among the 46 lymph nodes classified as indeterminate on preoperative CT, histopathological examination confirmed 35 as benign (76.09%) and 11 as metastatic (23.91%). Similarly, of the 38 lymph nodes deemed indeterminate on MRI, 29 were benign (76.32%) and 9 were metastatic (23.68%). In contrast, PET-CT classified only four lymph nodes as indeterminate (mean SUVmax, 3.43), all of which were histopathologically benign (100%), indicating a distinct diagnostic pattern compared with CT and MRI (Table 6).

### 3.2. LN Size & SUVmax Analysis

Comparison of lymph node (LN) characteristics across imaging modalities demonstrated significant differences between benign and metastatic LNs (Table 7). On CT imaging, metastatic LNs showed a significantly larger mean size (1.47 ± 0.53 cm) than benign LNs (1.06 ± 0.19 cm; t = 2.04, *p* = 0.046).

On PET-CT, metastatic LNs exhibited significantly higher SUVmax values compared with benign LNs (4.52 ± 2.44 vs. 1.11 ± 2.15, *p* < 0.001). Among LNs initially categorized as indeterminate on imaging, histopathologically metastatic nodes demonstrated higher SUVmax values than benign nodes (4.87 ± 1.74 vs. 3.01 ± 1.34; t = 4.79, *p* < 0.001). These findings indicate that both CT-measured LN size and PET-CT SUVmax are useful parameters for differentiating metastatic from benign lymph nodes in patients with OSCC.

### 3.3. SUVmax of Indeterminate Nodes

**Figure 3:**
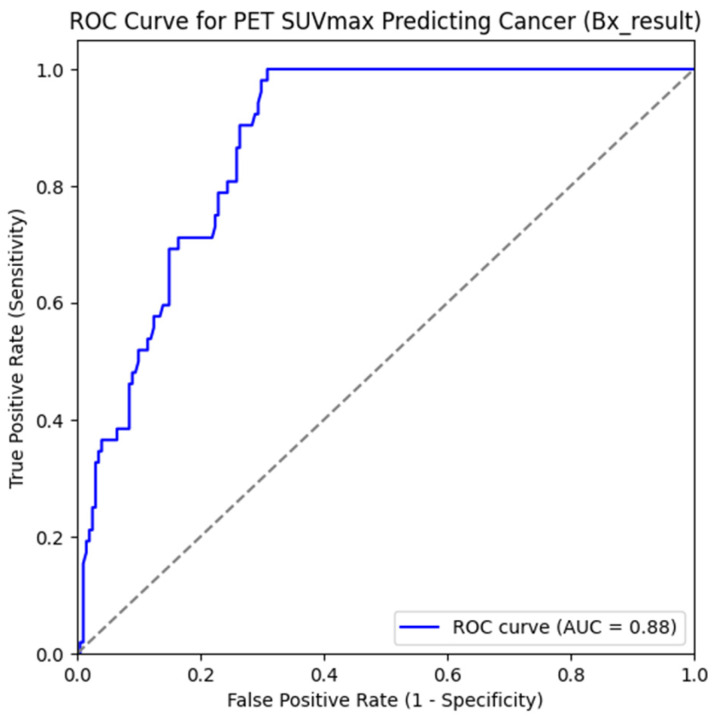
Receiver Operating Characteristic (ROC) analysis for SUVmax in predicting malignancy. The ROC curve demonstrated strong discriminatory performance, with an Area Under the Curve (AUC) of 0.88. The optimal cut-off value for malignancy prediction was 0.11, corresponding to an SUVmax threshold of 3.6. Logistic regression analysis showed statistical significance (*p* < 0.001), with an odds ratio of 1.71 (95% confidence interval: 1.48–2.35).

To evaluate the utility of PET/CT in further characterizing indeterminate lymph nodes, a Receiver Operating Characteristic (ROC) curve analysis was performed based on SUVmax values. The analysis showed a strong discriminatory ability for predicting malignancy, with an Area Under the Curve (AUC) of 0.88, indicating excellent diagnostic performance (Figure 3). Using logistic regression analysis, an optimal SUVmax cutoff value of 3.6 was identified. Nodes with SUVmax values above this threshold were significantly more likely to be malignant. This cutoff point provided a favorable balance between sensitivity and specificity, as demonstrated by the ROC curve. These findings support the use of SUVmax > 3.6 as a reliable threshold to differentiate metastatic from non-metastatic indeterminate lymph nodes on PET/CT, reinforcing its role in refining diagnostic accuracy when conventional imaging yields inconclusive results.

## 4. Discussion

This retrospective study evaluated the diagnostic performance of CT, MRI, and PET-CT for the detection of metastatic lymph nodes in patients with OSCC, with particular emphasis on lymph nodes classified as indeterminate on preoperative imaging. Unlike previous studies, the present analysis specifically focuses on this diagnostically challenging subgroup. By retrospectively correlating imaging findings with histopathological confirmation, the clinical significance of indeterminate imaging classifications was clarified.

Histopathological analysis showed that metastatic lymph nodes occurred more commonly in levels I and II of the neck and significantly less frequently in level III compared to benign nodes. This distribution may reflect the typical lymphatic spread pattern of OSCC, which predominantly involves cervical lymph node levels I and II [11,12]. Although the mean age was slightly higher in the metastatic group (67.88 years) than in the benign group (65.08 years), this difference was not statistically significant.

MRI-based assessment revealed that necrotic change and multifocality were more frequently associated with metastatic lymph nodes, whereas features such as unclear boundaries, cystic changes, and microcalcifications were uncommon but, when present, tended to support a diagnosis of malignancy [4,9,10,13]. In contrast, benign and indeterminate lymph nodes were often solitary and lacked necrotic or cystic changes, suggesting a lower likelihood of metastatic involvement.

These findings show the limitations of relying on a single imaging modality for the definitive diagnosis of lymph node metastasis. Certain reactive, non-inflammatory lymph nodes may present with enlargement and a rounded morphology, mimicking malignant features, while early-stage metastatic nodes may retain an oval shape and evade detection based on shape criteria alone on CT or MRI [14,15]. Therefore, additional imaging characteristics—such as thickening or epithelial layer enlargement, absence of the fatty hilum, and alterations in nodal vascularity—should also be considered [6,10,16]. In PET-CT, false-positive uptake may occur due to inflammatory or infectious processes, while micrometastatic disease may result in false-negative findings because of limited spatial resolution or low metabolic activity. These limitations highlight the need for integrated interpretation across modalities. The increasing development and advancement of multimodal imaging strategies, integrating ultrasound or digital PET/CT with anatomical imaging, may improve diagnostic accuracy by capturing a broader spectrum of morphological and functional changes associated with lymph node metastasis [1,2].

All three imaging modalities demonstrated high overall accuracy (>80%) for metastatic lymph node detection on initial assessment. CT showed the highest specificity (92.39%), reflecting its conservative diagnostic nature and low false positive rate, consistent with previous reports [1,5,8,14,17,18,19]. MRI showed slightly higher sensitivity but lower specificity, likely due to its superior soft tissue contrast, which may increase false-positive interpretation in reactive nodes, as reported by Thariat et al. [20] PET-CT demonstrated high sensitivity for excluding malignancy when SUVmax was low but showed variable specificity, in agreement with prior meta-analyses in head and neck squamous cell carcinoma [21]. When correlated with histopathology, radiologically indeterminate lymph nodes demonstrated distinct modality-specific diagnostic tendencies. Most indeterminate nodes identified on CT and MRI were ultimately benign, whereas a smaller proportion proved metastatic. In contrast, PET-CT classified very few nodes as indeterminate, all of which were histopathologically benign, with a mean SUVmax of 3.43. This distribution suggests that PET-CT more readily reclassifies equivocal nodes into benign or malignant categories, while CT and MRI more frequently retain indeterminate assessments (Table 6).

Quantitative analysis further demonstrated that metastatic lymph nodes were larger and exhibited significantly higher SUVmax values than non-metastatic nodes. For indeterminate nodes on CT, the metastatic group had a mean size of 1.22 cm compared to 1.07 cm for benign nodes (*p* = 0.046). Rasmussen et al. further validated a CT-based scoring system that enhances predictive accuracy for lymph node metastasis [6]. This finding was also consistent with Som’s identification of maximal diameter of 8 to 15 mm as a malignancy indicator in ambiguous nodes [14]. Similarly, SUVmax values were significantly elevated in metastatic nodes on PET-CT (*p* < 0.001), corroborating prior studies [22,23,24,25]. ROC analysis supported the discriminatory value of SUVmax, with an AUC of 0.84–0.88, and logistic regression demonstrated a significant association between increasing SUVmax and metastatic risk. These results are consistent with findings reported in prior studies, demonstrating the prognostic utility of SUVmax in differentiating metastatic from benign lymph nodes in patients with OSCC [1,2,21,24,26]. Although an SUVmax threshold of approximately 2.5 has been widely used to define hypermetabolic lymph nodes, this cutoff may be suboptimal in the evaluation of indeterminate nodes, in which inflammatory or reactive changes are common. Consistent with this, the four lymph nodes classified indeterminate on PET-CT in the present study demonstrated a mean SUVmax of 3.4 and were all histopathologically confirmed as benign. In this context, the higher ROC-derived cutoff identified in this study (SUVmax = 3.6) was associated with improved specificity, suggesting that SUVmax interpretation may benefit from contextual adjustment rather than reliance on a universal threshold. By incorporating histopathological confirmation of radiologically indeterminate lymph nodes, the present findings support the potential value of SUVmax-based analysis in refining diagnostic confidence when conventional imaging findings are inconclusive.

Overall, these findings indicate that a multimodal imaging approach may be beneficial, integrating CT for specificity, MRI for anatomical detail, and PET-CT for metabolic assessment, particularly in the evaluation of indeterminate lymph nodes in OSCC. Future prospective, multicenter studies incorporating advanced imaging techniques such as PET/MRI and diffusion-weighted imaging may further improve diagnostic accuracy and staging precision.

## 5. Conclusions

This study demonstrates that CT, MRI, and PET-CT each provide distinct and complementary diagnostic information for the assessment of cervical lymph node metastasis in OSCC, particularly in radiologically indeterminate cases. While CT offers high specificity and MRI provides enhanced anatomical detail, PET-CT contributes valuable metabolic information that facilitates reclassification of equivocal lymph nodes. Importantly, correlation with histopathological outcomes revealed that most indeterminate nodes on CT and MRI were benign, whereas PET-CT classified fewer nodes as indeterminate, suggesting different diagnostic thresholds among modalities. Quantitative PET-CT analysis further indicated that SUVmax may aid in refining diagnostic confidence in indeterminate lymph nodes, although universally applied cutoff values may be suboptimal. Collectively, these findings highlight the clinical value of a multimodal imaging strategy rather than reliance on a single modality for preoperative nodal evaluation in OSCC.

## Figures and Tables

**Table 1:** Diagnostic classification of each modality.

CT	Size	Nodes ≥ 15 mm
	Morphology	Necrosis, cystic change, calcification, or hyperenhancement
	Shape and margins	Infiltrating nodal tissue, ill-defined irregular margins
	Distribution	Asymmetrically prominent nodes or three or more contiguous
MRI	Intensity	Low T1-weighted and T2 hyperintensity
PET-CT	SUVmax	SUVmax ≥ 2.5

**Table 2:** CT Characteristic findings of the study subjects. * L/T(Long-axis(longitudinal) ÷ short-axis (transverse)).

Group	Benign LN (n = 164)	Indeterminate LN (n = 46)	Metastatic LN (n = 43)
**Multifocality**			
Single	143	22	41
Multiple	21	24	14
**Unclear boundary**			
Present	0	0	1
Absent	164	46	42
**Microcalcification**			
Present	0	0	0
Absent	164	46	43
**Cystic change**			
Present	0	0	2
Absent	164	46	41
**Necrotic change**			
Present	1	0	10
Absent	165	46	33
L/T *			
>2	10	4	3
≤2	154	42	40

**Table 3:** MRI Characteristic findings of the study subjects.

Group	Benign LN (n = 164)	Indeterminate LN (n = 38)	Metastatic LN (n = 43)
**Multifocality**			
Single	144	17	34
Multiple	20	21	9
**Unclear boundary**			
Present	0	0	2
Absent	164	38	41
**Microcalcification**			
Present	0	0	0
Absent	164	38	42
**Cystic change**			
Present	1	0	0
Absent	163	38	42
**Necrotic change**			
Present	0	0	7
Absent	164	38	36

**Table 4:** Histopathological result by sex and level of the study subjects.

Group	Benign LN (n = 192)	Metastatic LN (n = 52)
**Sex**		
Female	17	17
Male	23	16
**Age (years)**	65.08	67.88
**Level**		
I	50	22
II	50	22
III	69	4
IV	15	2
V	15	2

**Table 5:** Calculation used to measure Accuracy, Sensitivity, Specificity of CT/MRI/PET-CT.

Accuracy	Sensitivity	Specificity
(TP + TN)/(TP + FP + FN + TN) × 100	TP/(TP + FN) × 100	TN/(FP + TN) × 100

**Table 6:** Histopathological Outcomes of Lymph Nodes Classified as Indeterminate by CT, MRI, and PET-CT.

Modality	Indeterminate	Benign (n)	Metastatic (n)	Benign (%)	Metastatic (%)
**CT**	46	35/46	11/46	76.09%	23.91%
**MRI**	38	29/38	9/38	76.32%	23.68%
**PET**	4	4/4	0/4	100%	0%

**Table 7:** Mean CT-measured lymph node size and PET-CT SUVmax values according to histopathological diagnosis. Lymph nodes initially classified as indeterminate on preoperative imaging were further stratified based on final histopathological confirmation. * *p* < 0.05.

Initial Radiologic Report	Benign	Metastatic	Indeterminate	
**Histopathologic confirmation**	Benign	Metastatic	Benign	Metastatic
CT (cm) *	1.06 ± 0.19	1.47 ± 0.53	1.07 ± 0.37	1.22 ± 0.36
**PET-CT** (SUVmax) *	1.11 ± 2.15	4.52 ± 2.44	3.01 ± 1.34	4.87 ± 1.74

## Data Availability

The data supporting the findings of this study are not publicly available due to ethical restrictions imposed by the institutional review board to protect patient privacy.

## Abbreviations

The following abbreviations are used in this manuscript:

AUC	Area Under the Curve
CI	Confidence Interval
CT	Computed Tomography
DWI	Diffusion-Weighted Imaging
FDG	Fluorodeoxyglucose
LN	Lymph Node
L/T	Long-to-Short Axis Ratio
MRI	Magnetic Resonance Imaging
mRND	modified Radical Neck Dissection
OR	Odds Ratio
OSCC	Oral Squamous Cell Carcinoma
PET	Positron Emission Tomography
PET-CT	Positron Emission Tomography–Computed Tomography
PNUDH	Pusan National University Dental Hospital
ROC	Receiver Operating Characteristic
SOHND	Supraomohyoid Neck Dissection
SPSS	Statistical Package for the Social Sciences
SUVmax	Maximum Standardized Uptake Value
